# A Preliminary Investigation of the Effects of Obstacle Negotiation and Turning on Gait Variability in Adults with Multiple Sclerosis

**DOI:** 10.3390/s21175806

**Published:** 2021-08-28

**Authors:** Lara Weed, Casey Little, Susan L. Kasser, Ryan S. McGinnis

**Affiliations:** 1 Department of Electrical and Biomedical Engineering, University of Vermont, Burlington, VT 05405, USA; laraweed@stanford.edu; 2 Department of Rehabilitation and Movement Science, University of Vermont, Burlington, VT 05405, USA; casey.little@uvm.edu (C.L.); susan.kasser@med.uvm.edu (S.L.K.)

**Keywords:** wearables, gait, variability, multiple sclerosis, mobility, inertial sensors, walk, turn, obstacle crossing, sensor fusion, torso, spatiotemporal, falling

## Abstract

Many falls in persons with multiple sclerosis (PwMS) occur during daily activities such as negotiating obstacles or changing direction. While increased gait variability is a robust biomarker of fall risk in PwMS, gait variability in more ecologically related tasks is unclear. Here, the effects of turning and negotiating an obstacle on gait variability in PwMS were investigated. PwMS and matched healthy controls were instrumented with inertial measurement units on the feet, lumbar, and torso. Subjects completed a walk and turn (WT) with and without an obstacle crossing (OW). Each task was partitioned into pre-turn, post-turn, pre-obstacle, and post-obstacle phases for analysis. Spatial and temporal gait measures and measures of trunk rotation were captured for each phase of each task. In the WT condition, PwMS demonstrated significantly more variability in lumbar and trunk yaw range of motion and rate, lateral foot deviation, cadence, and step time after turning than before. In the OW condition, PwMS demonstrated significantly more variability in both spatial and temporal gait parameters in obstacle approach after turning compared to before turning. No significant differences in gait variability were observed after negotiating an obstacle, regardless of turning or not. Results suggest that the context of gait variability measurement is important. The increased number of variables impacted from turning and the influence of turning on obstacle negotiation suggest that varying tasks must be considered together rather than in isolation to obtain an informed understanding of gait variability that more closely resembles everyday walking.

## 1. Introduction

Multiple sclerosis is a chronic, progressive neurodegenerative disease that affects approximately 2.3 million people worldwide and is characterized by the demyelination of neurons disrupting the conduction of electrical impulses [1]. Persons with multiple sclerosis (PwMS) experience a variety of symptoms that can be unpredictable, vary in type and severity, and may be relapsing and remitting [2]. Some common symptoms experienced by PwMS include fatigue, weakness, poor coordination, imbalance, numbness, tingling, vision, cognitive problems, and pain [2]. Consequentially, approximately 75–90% of PwMS experience mobility issues [3] and PwMS fall at a higher rate and more often than those aging without neurologic impairment [4]. Over half of PwMS fall in any 6-month period [5], with many experiencing repeated falls and falls-related injury [6]. Falls can intensify already existing fear of falling [7], decrease physical activity [6], and reduce quality of life [8].

In PwMS, a large proportion of falls occur while walking and turning [9]. Compared to individuals without MS, PwMS demonstrate increased trunk range of motion and turning duration [10]. Turning has emerged as an important predictor of perceived balance confidence and ambulation disability in this group [11]. In addition to walking and turning, tripping when negotiating obstacles is also a common cause of falls. Research has shown that trips and slips while walking account for almost half of falls in PwMS [12]. In fact, the odds of a fall compared to a near-fall are five times more likely due to tripping over an obstacle [13]. This is especially important as, relative to non-fallers, fallers with MS poorly control their center of mass over their base of support and their foot swing is closer to the ground when stepping [14]. While Santinelli et al. [15] have examined gait during obstacle avoidance in PwMS, the gait changes they found between unobstructed walking and obstacle avoidance only included the leading and trailing step during crossing. Understanding how PwMS approach and continue walking after higher risk daily tasks is relevant and ecologically important. Yet, turning and preparing to cross obstacles are rarely examined empirically or qualitatively considered in clinical assessment of this population.

Maintaining the postural control and stability necessary to successfully turn or navigate an obstacle requires effective planning and real-time monitoring and adjustments. Research has shown that older adults not only turn less effectively but also have more gait speed and step-to-step variability when approaching an obstacle [16]. While preparatory gait adjustments are important to safely turn or negotiate an obstacle, high variability affects stability and increases the potential of falling. Increased gait variability is prominent in PwMS [17] and serves as a robust predictor of falls [14,18]. However, it is unclear the extent to which movement variability exists in the context of turning or obstacle negotiation. Additionally, gait variability is typically quantified by considering the variability in spatial or temporal gait parameters [18], but little is known about the variability in movement of other body segments (e.g., the torso) that impact stability during walking. Given that movement of the lower limbs and torso both impact the positioning of an individual’s center of mass relative to their base of support, it is critical that we consider variability of both the lower limbs and torso when investigating the relationship between movement variability and fall risk. Similarly, it is also problematic that many clinical measures involve straight line gait assessment and typically do not include contexts reflective of real-world walking where changes of direction and obstacle negotiation are common.

Wearable sensor-based approaches provide the opportunity to understand real-world gait and mobility patterns in PwMS [18]. The advancement of inertial measurement units (e.g., size, weight, power) and algorithms (for example, see [19,20,21,22,23]) enable assessments of biomechanical parameters such as those measured in the criterion standard camera-based motion capture system but without limitations due to capture space, marker occlusion, and laboratory setting limitations that may make it more difficult to calculate variability [24]. In previous studies, wearable devices have been used to quantify straight line walking gait variability and longer duration bouts such as during the 6-min walk test [24]. However, there have been few studies on gait variability in the negotiation of obstacles and turning, most of which have focused on the negotiation steps [15] rather than the gait characteristics preceding and succeeding obstacles and turns.

Developing a more detailed understanding of variability that may put PwMS at greater fall risk when walking under different conditions is therefore needed. As such, the purpose of this study was to examine gait variability before and after obstacle negotiation and with turning in PwMS. Such insight has the potential to inform clinical assessment and improve targeted interventions that could improve postural stability and balance when walking.

## 2. Materials and Methods

### 2.1. Participants

Informed consent was obtained from PwMS (N = 12, 8F, Age: 61.4 ± 8.7) and age- and sex-matched heathy controls (N = 11, 8F, Age: 59.9 ± 8.7). PwMS were eligible to participate if they had physician-diagnosed MS and self-report minimal to moderate disability (0 to 4 on the Patient Determined Disease Steps scale [25]). Matched control participants were eligible if neuro-typical without a history of brain injury, neurological impairment, psychiatric disorder, or learning disability. Eligibility was determined through telephone screening and written informed consent was obtained prior to data collection. This project was reviewed and approved by the local Institutional Review Board.

### 2.2. Sample Descriptive Measures

Clinical measures of mobility, falling, and fatigue were assessed for PwMS. Perceived mobility limitations and ambulation disability were assessed with the multiple sclerosis walking scale-12 (MSWS-12) [26], which is reliable and valid [27]. The falls efficacy scale—international (FES-I) [28] measured each participant’s concerns about falling when performing seven daily activities. The FES-I is reliable, valid, and predictive of fall risk in PwMS [29]. The effect of perceived fatigue on the participant’s functioning was assessed using the modified fatigue impact scale (MFIS) [30], which is valid and associated with balance deficits and falls in PwMS [31].

Table 1 provides descriptive details for all participants. Of the PwMS, the majority self-report moderate ambulation disability (PDDS 2–4). On average, the participants were middle-aged and have been living with MS for over two decades. They had moderate fatigue and moderate to high concerns about falling.

### 2.3. Procedures

**Figure 1:**
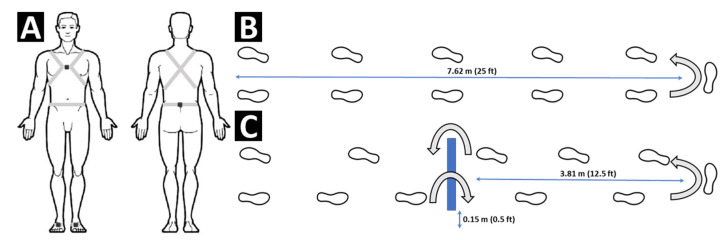
Inertial sensors placement (**A**) at the bilateral feet, chest, and lumbar spine and the sample courses for the walk and turn task (**B**) and walk and turn with obstacle task (**C**). For both tasks, the length of the course was 7.62 m (25 ft). The obstacle in the walk and turn with obstacle was placed at approximately 3.81 m (12.5 ft) from the starting position and was 0.15 m (0.5 ft) tall.

Participants were instrumented with OPAL wireless inertial sensors (ADPM Inc., Portland, OR, USA) secured with straps to the medial chest, lumbar spine, and bilateral feet, which can be seen in Figure 1A. Data from the OPAL sensors were collected wirelessly at 128 Hz onto a desktop computer.

After performing a standing calibration, participants completed two walking trials: (1) A walk and turn (WT) in which they walked forward 7.62 m, turned 180 degrees, and returned to the start, and (2) a walk and turn with obstacle (OW), in which they walked the 7.62 m path stepping over a 0.15 m high obstacle placed midway, turning 180 degrees, and again negotiating the obstacle on their way back to the start, which can be seen in Figure 1B,C, respectively. Each individual self-selected their pace after being instructed to walk at a purposeful, yet safe, pace with their arms swinging naturally by their sides. A research assistant moved alongside the participant to provide stand-by support and prevent the possibility of falling.

### 2.4. Data Analysis

Acceleration, angular velocity, and quaternion data were captured from each sensor and processed using custom MATLAB (Mathworks, Inc., Natick, MA, USA) scripts. Standing anatomical calibrations were manually selected during still periods before each trial and used to align the sensors with a body-fixed reference frame (z-axis: Cranial-caudal, x-axis: Anterior-posterior direction, y-axis: Mediolateral) following which temporal, spatial, and body parameters were extracted from each phase of each walking task.

#### 2.4.1. Temporal Parameters

**Figure 2:**
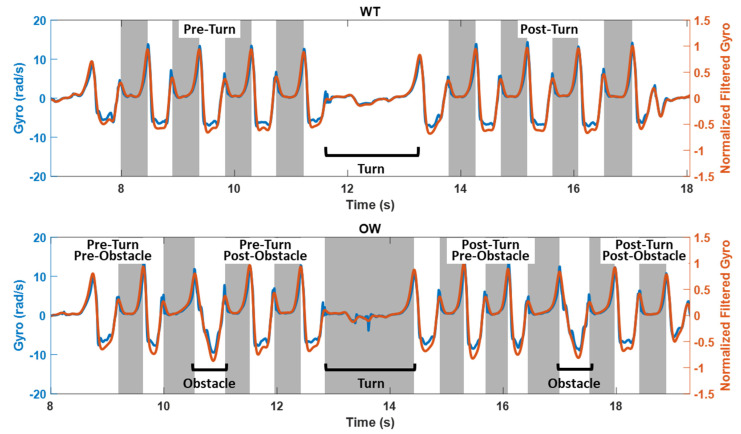
Example HS and TO detection from normalized filtered gyro from the right foot for the WT task (top) and the OW task (bottom). Raw mediolateral gyro data and normalized, filtered gyro data are in blue and orange, respectively. Gray shading marks HS to TO. Phases of the tasks, turning, and obstacle crossings are labeled. Note that detection of HS and TO were dependent on the expected peaks in the filtered and normalized mediolateral gyro signal.

Heel-strike (HS) and toe-off (TO) events were detected using an algorithm adapted for the feet from [32], which is typically used at the shank. Mediolateral angular velocity from the bilateral feet was lowpass filtered (2nd-order butterworth; 6 Hz cutoff frequency) and used to identify the swing phase of each gait cycle. Normalization by the maximum absolute value of the foot gyro signal was used to account for greater subject to subject variability in the peak detection at the foot location compared to the shank. The last peak before the swing was then identified as TO and the first peak after the swing was identified as HS. An example of HS and TO detection from filtered, normalized gyro data can be seen in Figure 2 for both the WT and OW tasks. The HS-TO detection was used to segment steps from TO to TO and calculate the temporal gait parameters cadence, step time (TO-TO), and contact time (HS-TO). Note that TO was used to segment strides, rather than the more common HS, as this approach was able to accommodate transitional strides directly before and after the obstacles and turns.

#### 2.4.2. Spatial Parameters

At the bilateral feet, a pedestrian tracking sensor fusion algorithm was used to provide estimates of the position and velocity of each foot in an earth-fixed reference frame [19]. This method leverages the fact that there is at least one instant in time when the foot has zero velocity when it is in contact with the ground, also known as a zero-velocity update (ZUPT). Here, ZUPTs were estimated by considering the difference in angular velocity and acceleration relative to data recorded during a quiet standing period prior to the start of each task. When this difference was below a threshold determined based on the distribution of data in the quiet standing period, a ZUPT was identified. The position and velocity estimated for each foot using the pedestrian tracking algorithm was used to calculate the step length, step clearance, lateral deviation (lateral range of step including swing), foot attack angle (arctangent of vertical and forward velocity vectors upon heel-strike), and maximum foot velocity during the swing were extracted for each step and foot.

#### 2.4.3. Body Parameters

Sensors located on the trunk and lumbar were used to calculate body parameters for each step as segmented by the HS-TO detection. Using the quaternions produced by APDM, yaw parameters were calculated by projecting the change in angle between quaternions at consecutive timepoints onto the yaw direction. Yaw range and the average rate of change during a step, defined as yaw rate, were calculated to characterize the rotational motion of trunk and lumbar.

#### 2.4.4. Phase Classification

The WT task was divided into two phases, pre-turn and post-turn, based on where a step occurred relative to the turn. Turning steps were identified as steps in which the trunk had a yaw range of greater than 100 degrees. Turning steps, and their associated gait parameters, were excluded from further analysis.

**Figure 3:**
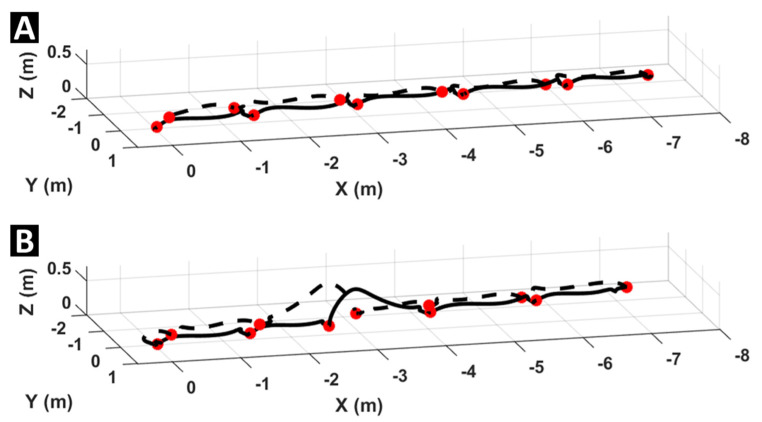
Step trajectories from one foot during the walk and turn task (**A**) and the walk and turn with obstacle task (**B**) from a representative participant from the MS group. Toe-off timepoints are indicted with red dots and dashed lines indicate trajectories following the turn in each task.

The OW task used the same methods to classify pre-turn and post-turn phases but also included phases before and after each obstacle for a total of four distinct phases (i.e., pre-turn pre-obstacle, pre-turn post-obstacle, post-turn pre-obstacle, and post-turn post-obstacle). Obstacle negotiation steps were defined as the step with the greatest step height for each leg during the pre-turn and post-turn phases. Obstacle steps, and their associated gait parameters, were excluded from further analysis. Representative foot trajectories during the WT and OW tasks of a PwMS can be seen in Figure 3A,B, respectively.

#### 2.4.5. Gait Variability Calculation

For each phase in the two tasks, stride-to-stride variability of the parameters was calculated and used for analysis. Variability was defined as the mean absolute difference between steps within a phase and was calculated using the following equation:

(1) $$\mathrm{Gait}\;\mathrm{Variability}=\frac{\sum_{i=2}^{n}\left|x_{i}-x_{i-1}\right|}{n-1},$$

where *x* is the value of the parameter and *n* is the number of steps in the phase. For each task, differences in variability parameters of the phases within PwMS and between PwMS and controls were examined. This definition of variability was used rather than the more traditional measures of variance (e.g., standard deviation) to capture inter-step dynamics due to the limited and variable number of steps in each phase of the task. Recent evidence suggests that gait characteristics estimated from shorter walking bouts may be more informative in quantifying the disability level, emphasizing the need for alternative measures of gait variability that can be computed reliably when a limited number of strides are available for analysis [3].

#### 2.4.6. Statistical Analysis

Three PwMS were excluded from the analysis due to data collection error. As a result, N = 9 PwMS (5F, Age: 63.1 ± 7.2 years) and N = 11 heathy controls were included in the within-group analyses. For the between-group analyses, eight PwMS (5F, Age: 62 ± 6.8 years) and eight age- and sex-matched healthy controls (5F, Age: 61.3 ± 7.7 years) were considered. Specifically, for the WT task, the impact of turning on gait variability parameters for each group and the impact of MS on the turn approach and recovery were examined. For the OW task, the impact of the obstacle before and after the turn, the impact of the turn on approaching and recovering from an obstacle, and the impact of MS on the negotiation of each phase were examined.

A Lilliefors test of normality was performed on each phase and variability parameter for the two groups. The parameters did not follow a normal distribution, and therefore, a Wilcoxon rank-sum test was selected to evaluate significant differences in each comparison. A significance level of 0.05 was used to determine significant differences. To quantify the effect size of significant differences, a rank-biserial correlation was calculated and given a sign (±) to indicate the direction.

## 3. Results

We first consider the Walk and Turn task by examining the difference in the variability of the temporal and spatial foot and body metrics between the pre- and post-turn phases in PwMS and controls. Table 2 and Table 3 report mean variability, *p*-value (*p*), and effect size (*r*) for each comparison for PwMS and controls, respectively. PwMS and controls both exhibit significantly less variability in lumbar and trunk yaw range and trunk yaw rate in the pre-turn phase. PwMS also demonstrate significantly less variability in lumbar yaw rate, lateral foot deviation, cadence, and step time indicating lower variability in temporal, spatial, and body-based parameters. Directly comparing the two groups, PwMS exhibit significantly lower step length, lumbar yaw range, and lumbar yaw rate variability during the pre-turn phase than controls.

To explore the impact of a more challenging ambulation task on gait variability, the differences between the phases of the Obstacle Walk were examined next. Specifically, four comparisons were considered: (1) Pre-turn pre-obstacle to pre-turn post-obstacle (pre-turn), (2) pre-turn pre-obstacle to post-turn pre-obstacle (pre-obs), (3) pre-turn post-obstacle to post-turn post-obstacle (post-obs), and (4) post-turn pre-obstacle to post-turn post-obstacle (post-turn). Table 4 and Table 5 report mean variability, *p*-value (*p*), and effect size (*r*) for each comparison for PwMS and controls, respectively.

For PwMS, significant differences are observed in the pre-turn, pre-obs, and post-turn comparisons. For pre-turn, PwMS demonstrate significantly more variability during the pre-obstacle phase in both spatial and temporal gait parameters. Interestingly, in the post-turn comparison, PwMS exhibit significantly more variability during the pre-obstacle phase in spatial, temporal, and body-based measures. In the pre-obs comparison, PwMS exhibit significantly more variability in spatial gait measures in the pre-turn phase than the post-turn phase, but less variability in body-based measures.

For controls, significant differences are observed in all four comparisons. In the pre-turn comparison, the controls demonstrate more temporal variability and less body variability during the pre-obstacle phase. In the pre-obs comparison, controls exhibit significantly less variability during the pre-turn pre-obstacle phase in spatial, temporal, and body-based measures than in the post-turn pre-obstacle phase. Notably, these same differences in temporal variability are not present in PwMS and the differences in spatial variability are in the opposite direction of those observed in PwMS. Finally, in the post-turn comparison, controls exhibit significantly more variability during the post-turn post-obstacle phase in the temporal, spatial, and body-based measures than in the post-turn pre-obstacle phase.

Examining the differences between PwMS and controls during each phase of the obstacle walk, significant differences were observed in temporal, spatial, and body-based variability measures (Table 6). During the pre-turn pre-obstacle phase, PwMS have significantly more contact time variability than controls. During the pre-turn post-obstacle phase, PwMS have significantly less lumbar and trunk yaw rate variability than controls. Finally, during the post-turn pre-obstacle phase, PwMS have significantly less step clearance variability than controls.

## 4. Discussion

Falls are a major problem for PwMS making the early identification and treatment of those at risk essential. Gait variability, while partially reflective of the quality and functionality of movement control [33], is of greater magnitude in PwMS [10,17] and closely linked to falls [14,34,35]. However, most studies that examine gait variability in PwMS assess those more mildly impaired [36] and measure spatiotemporal parameters during straight line ambulation, which is less reflective of real-world walking contexts. The unique aspect of our study was the investigation of gait variability while walking and turning as well as walking and crossing an obstacle using body-worn motion sensors to examine both spatiotemporal parameters and body-based measures.

Consistent with previous research [10,35,37], PwMS in this study had significant variability across multiple gait metrics compared to healthy controls, including step length and trunk motion. These results are further discussed in the sections below.

### 4.1. Turning

Changes in direction represent an important aspect of daily mobility yet are often overlooked in gait analysis in PwMS. The few studies that have examined turning have shown compromised and quantitatively different turn behavior in PwMS compared to those without MS [38]. Additionally, while turns have been included in some empirical studies, the quality of gait after a turn is not well understood. In fact, when comparing straight walking with walking and turning, turning velocity improved the predictive power of traditional clinical tests of gait speed [11].

In the present study, the measurement of gait variability was indeed found to be affected by turning. PwMS demonstrated significantly more variability in lumbar and trunk yaw range and rate, lateral foot deviation, cadence, and step time after the turn compared to before. Control participants, on the other hand, while exhibiting significantly more variability in lumbar and trunk yaw range and trunk yaw rate post-turn versus pre-turn walking, did not show any significant differences in the spatiotemporal parameters measured. This may indicate that turning perturbed the PwMS and required more recovery steps contributing to the significant differences pre- versus post-turn, whereas the control subjects were able to recover more quickly.

Maintaining balance during and after turning involves the complex coordination of sensory and motor systems needed to stabilize and reorient the body towards the new direction of travel. When moving through a turn to resume straight line walking, visual, vestibular, and proprioceptive inputs must be reweighted for effective postural orientation and stability [39]. While a range of underlying mechanisms contribute to gait variability in PwMS [35], the varying stride characteristics observed after turning suggest the ineffectiveness of the balance control system to quickly adapt to the requirements of the turn. As suggested in previous MS research on turning, the variability in gait observed in our study may suggest an impaired sensory integration system necessary to quickly reorient the head, trunk, and pelvis [10]. It can be speculated that MS-related deficits in sensory inputs and integration, along with base of support and force generation limitations often found in PwMS [40], combine to not only decrease efficiency in balance control but also destabilize gait after the turn when resuming straight line ambulation. It is likely that the control participants were better able to more quickly adapt and reorient to the turn, either while turning or within a stride or two after, while those with MS needed multiple steps and longer to adapt and recover.

The fact that there were no significant variability differences found between PwMS and controls after turning may be attributed, in part, to the small sample size. However, the large effect sizes found may also suggest that individuals simply adapt to turning perturbations differently. It would be important to more deeply and qualitatively examine gait stability strategies to further our understanding of differences between individuals with and without MS.

### 4.2. Obstacle Negotiation

When comparing walking in the context of obstacle negotiation, PwMS exhibited more variability in both spatial and temporal gait parameters before crossing the obstacle compared to after. While both the PwMS and control groups slowed down when approaching the obstacle, PwMS had significantly greater contact time variability and also had significant variability in step length, cadence, and other spatiotemporal parameters in order to prepare for stepping over the obstacle that was not observed in control participants. The control group also showed significantly greater variability in lumbar yaw range and rate pre-obstacle versus post-obstacle that was not evident in PwMS. While speculative, this variability in body-based measures by controls may be indicative of their ability to adaptively control their center of mass in relation to their base of support when successfully approaching and negotiating an obstacle. Conversely, for PwMS, it may be that their shorter and more variable step length and slower gait speed ensured minimal requirement for torso adjustment in order to maintain stability of the upper body for this task [41], or they lacked the needed variability in trunk movement for optimal balance control given their varying spatial and temporal performance, thus potentially placing them at greater risk for trips or falls. Moreover, the pre-crossing phase of obstacle negotiation requires visually-dependent motor planning for step regulation that is attentionally demanding [42]. As cognitive processing impairments may have a negative impact on postural activity and anticipatory gait control before crossing an obstacle [15,43], the deficits in attention and executive function found in PwMS [44] may be a potential cause of trip-related falls in this population and warrants further exploration.

Not only does crossing an obstacle increase balance demands of the sensorimotor system, but it also appears that turning before negotiating an obstacle further impacts variability and balance control. When assessing the effect of turning on preparing to cross an obstacle, PwMS were more variable in temporal gait measures but less variable in body-based measures post-turn than pre-turn. Control participants also exhibited significantly more variability after turning compared to before turning, although in many more spatial, temporal, and body-based parameters. Compared to control participants, though, PwMS had significantly less lumbar and trunk yaw rate variability after crossing the obstacle but before turning compared to completing the turn and then walking toward the obstacle. The findings indicate that turning further impacted straight line walking when readying to cross an obstacle and, as previously suggested, the lack of body-based variability in PwMS post-turn may limit their recovery from making the turn and negatively influence their ability to successfully approach and negotiate the upcoming obstacle. PwMS also seemed to move more slowly and carefully than controls.

The greatest number of significant differences in walking with an obstacle in the path were found after turning. There was more spatial, temporal, and body-based variability found immediately after the turn before crossing the obstacle compared to after crossing the obstacle for both the PwMS and control groups. It may be that the attentional demands required by the two tasks, turning and readying for obstacle crossing, are compounded and the requisite ability to task switch impacted [45]. Interestingly, for the PwMS there were no significant differences in variability of any parameters after crossing the obstacle, both before turning and after. It is likely that, by the time participants crossed the obstacle and continued in a straight line, there was enough time for reorienting the body and potentially decreasing the effects of the turn on variability and balance control. Controls, on the other hand, demonstrated the greatest extent of variability across parameters when approaching the obstacle before and after the turn as well as in pre- and post-obstacle walking after turning. It can be speculated that, while increased variability during straight line walking may increase risk of falls [46,47], reduced not increased variability—especially in body-based parameters—may decrease necessary gait adaptations when turning and approaching an obstacle and, thus, may also put PwMS at risk for trips or falls. However, future work is required to further explore the impact that these observations have on fall risk in PwMS.

### 4.3. Strengths, Limitations, and Future Work

Collectively, our findings suggest that the context of gait analysis in PwMS warrants consideration. Variability in straight line walking is differentially impacted by changing direction or preparing to cross an obstacle and these effects are compounded when doing both. Clinical assessment using straight line walking without examining gait variability as a performance metric, cannot provide the comprehensive understanding needed.

While the study was novel and our findings offer important clinical implications, some limitations exist. Our sample of PwMS was small and only included those who had mild to moderate walking impairment. As such, these findings are not representative of all PwMS. Future research with larger and more diverse cohorts are needed to confirm our findings and offer insight into the generalizability of these results. It would also be beneficial to investigate how gait variability in the context of turns and obstacle negotiation differs and changes over the course of the disease especially as increases in gait variability during straight line walking are more pronounced with disease progression [17]. It would also be important to examine how gait variability within these contexts is associated with falls, and to further consider potential confounding due to age. The present study also only involved a 180-degree-turn which does not fully represent the varying direction changes naturally occurring in daily living. Conducting research that examines the effects of different degrees of turning on gait stability and variability would allow a more ecologically valid understanding of gait. Additionally, our gait analysis was based on a relatively fewer number of steps, which may be more reflective of real-world bouts. It has been suggested that gait variability parameters require longer distances to demonstrate good reliability [48]. Future research is necessary to explore the differences in reliability using variability measures appropriate for shorter bouts such as those considered during the WT (average of 22 steps) and OT (average of 6–12 steps across the four phases) tasks reported here. Future research may also employ remote monitoring of daily ambulation and environmental navigation as a more ecologically valid way to measure gait variability and assess real-world fall risk. Finally, it would behoove researchers to develop and test clinical measures, inclusive of turns and obstacle crossings, that more objectively assess gait variability and offer a more holistic picture of mobility and fall risk.

## 5. Conclusions

This study investigated the kinematic nature of gait variability during walking before and after turning and obstacle negotiations. Results suggest that the context of the gait variability parameters are important in understanding differences that may contribute to fall risk in PwMS. Turning and obstacle negotiation seem to differentially impact gait variability which may be particularly important to consider when considering real-world circumstances. Limitations of this study included a relatively small sample size and limited range of mobility impairment. Future work should further explore the relationship of gait variability and fall risk in the context of real world environments through remote monitoring.

## Figures and Tables

**Table 1:** Sample demographics. Sample sizes (N) and mean (standard deviation) of variables are reported as appropriate. * Note that all participants are included with a subset of eight being age- and sex-matched. Within group analyses contained all participants whereas between group analyses contained only age- and sex-matched participants.

Variable	MS	Control
Sex (N)	Female: 8	Female: 8
	Male: 4	Male: 3
Phenotype (N)	Relapse-Remitting: 6	-
	Secondary Progressive: 6	-
PDDS (N)	0–1 (mild): 2	-
	2–4 (moderate): 10	-
Age *	61.4 (8.7)	59.9 (8.7)
Years post diagnosis	22.6 (15.7)	-
MSWS-12	43.5 (27.8)	-
FES-I	32.5 (12.4)	-
MFIS	32.3 (14.3)	-

PDDS: Patient determined disease steps; MSWS-12: Multiple sclerosis walking scale; MFIS: Modified fatigue impact scale; FES-I: Falls efficacy scale international.

**Table 2:** Comparisons of gait variability during phases of the walk and turn task (pre-turn—post-turn) for PwMS and controls (HC). *p*-values (*p*) are reported for each comparison with effect sizes (*r*) indicated for significant differences.

		WT Pre-Turn—Post-Turn					
		MS			HC		
	Gait Parameter	Pre-Turn Mean (STD)	Post-Turn Mean (STD)	*p*(*r*)	Pre-Turn Mean (STD)	Post-Turn Mean (STD)	*p*(*r*)
T	Cadence	**1.86 (1.78)**	**5.21 (5.66)**	**0.03 (−0.56)**	2.78 (2.93)	6.34 (7.31)	0.21
	Contact Time	0.03 (0.01)	0.05 (0.04)	0.07	0.02 (0.01)	0.04 (0.03)	0.12
	Step Time	0.03 (0.02)	0.08 (0.05)	0.02 (−0.56)	0.04 (0.03)	0.08 (0.07)	0.17
S	Foot Attack Angle	21.18 (24.44)	25.34 (31.77)	0.25	15.41 (11.76)	18.35 (13.72)	0.46
	Lateral Deviation	**0.02 (0.01)**	**0.03 (0.01)**	**0.05 (−0.82)**	0.03 (0.03)	0.04 (0.03)	0.37
	Max Swing Velocity	0.36 (0.12)	0.38 (0.17)	0.73	0.37 (0.09)	0.48 (0.22)	0.17
	Step Height	0.03 (0.02)	0.03 (0.02)	1	0.03 (0.01)	0.03 (0.01)	0.46
	Step Length	0.11 (0.05)	0.13 (0.07)	0.65	0.13 (0.03)	0.17 (0.08)	0.64
B	Lumbar Yaw Range	**2.32 (2.69)**	**20.11 (10.50)**	**<0.01 (−1.00)**	**5.59 (6.64)**	**20.48 (9.34)**	**0.05 (−0.67)**
	Trunk Yaw Range	**2.68 (2.24)**	**18.76 (8.27)**	**<0.01 (−1.00)**	**7.24 (5.38)**	**21.76 (3.78)**	**<0.01 (−0.88)**
	Lumbar Yaw Rate	**4.50 (2.71)**	**12.31 (13.47)**	**<0.01 (−1.00)**	6.82 (6.87)	14.75 (10.96)	0.07
	Trunk Yaw Rate	**3.57 (2.03)**	**12.32 (10.26)**	**<0.01 (−1.00)**	**5.20 (5.48)**	**14.35 (8.04)**	**<0.01 (−0.67)**

**Table 3:** Comparisons of gait variability during phases of the walk and turn task between PwMS and controls (HC). *p*-values (*p*) are reported for each comparison with effect sizes (*r*) indicated for significant differences.

		WT MS—HC					
		Pre-Turn			Post-Turn		
	Gait Parameter	MS Mean (STD)	HC Mean (STD)	*p*(*r*)	MS Mean (STD)	HC Mean (STD)	*p*(*r*)
T	Cadence	1.93 (1.88)	1.94 (1.87)	0.11	5.35 (5.96)	5.78 (6.64)	0.84
	Contact Time	0.03 (0.01)	0.02 (0.01)	0.55	0.05 (0.04)	0.04 (0.04)	0.46
	Step Time	0.03 (0.02)	0.03 (0.02)	0.25	0.08 (0.05)	0.09 (0.08)	0.74
S	Foot Attack Angle	23.50 (25.01)	14.22 (9.15)	0.43	27.15 (33.31)	18.06 (14.12)	0.7
	Lateral Deviation	0.02 (0.01)	0.03 (0.02)	0.38	0.03 (0.01)	0.04 (0.02)	0.55
	Max Swing Velocity	0.35 (0.13)	0.36 (0.06)	0.31	0.38 (0.18)	0.44 (0.21)	0.95
	Step Height	0.03 (0.02)	0.03 (0.01)	0.55	0.03 (0.02)	0.03 (0.02)	0.55
	Step Length	**0.11 (0.05)**	**0.13 (0.03)**	**0.05 (−0.89)**	0.12 (0.07)	0.15 (0.07)	0.38
B	Lumbar Yaw Range	**2.37 (2.84)**	**8.72 (7.20)**	**0.02 (−0.94)**	19.58 (10.28)	21.03 (9.61)	0.55
	Trunk Yaw Range	2.78 (2.34)	5.88 (6.04)	0.55	18.99 (8.69)	20.46 (3.82)	0.11
	Lumbar Yaw Rate	**3.25 (2.78)**	**11.07 (7.10)**	**0.04 (−0.72)**	19.30 (13.37)	20.00 (9.73)	0.74
	Trunk Yaw Rate	3.69 (2.07)	7.91 (6.06)	0.15	17.03 (10.81)	20.46 (6.57)	0.2

**Table 4:** Comparisons of gait variability during phases of the walk and turn with obstacle task for PwMS. *p*-values (*p*) are reported for each comparison with effect sizes (*r*) indicated for significant differences. Comparisons: Pre-turn pre-obstacle to pre-turn post obstacle (pre-turn), pre-turn pre-obstacle to post-turn pre-obstacle (pre-obs), pre-turn post-obstacle to post-turn post-obstacle (post-obs), and post-turn pre-obstacle to post-turn post-obstacle (post-turn).

		Mean (STD) Gait Variability				Pre-Turn	Pre-Obs	Post-Obs	Post-Turn
	Gait Parameter	Pre-Turn Pre-Obstacle	Pre-Turn Post-Obstacle	Post-Turn Pre-Obstacle	Post-Turn Post-Obstacle	*p*(*r*)	*p*(*r*)	*p*(*r*)	*p*(*r*)
T	Cadence	**5.37 (1.40)**	**3.31 (5.33)**	**6.37 (4.38)**	**2.36 (1.87)**	**0.01 (0.96)**	0.73	0.65	**0.01 (0.69)**
	Contact Time	**0.11 (0.03)**	**0.03 (0.02)**	**0.12 (0.09)**	**0.08 (0.14)**	**<0.01 (1.00)**	0.30	0.25	**<0.01 (−1.00)**
	Step Time	**0.11 (0.04)**	**0.04 (0.03)**	**0.12 (0.07)**	**0.05 (0.04)**	**<0.01 (1.00)**	0.73	0.57	**0.02 (0.64)**
S	Foot Attack Angle	**48.17 (35.85)**	**19.53 (20.76)**	35.84 (36.73)	28.47 (41.27)	**0.02 (0.60)**	0.10	0.13	0.82
	Lateral Deviation	0.03 (0.02)	0.03 (0.01)	0.03 (0.02)	0.02 (0.01)	0.82	0.50	0.73	0.10
	Max Swing Velocity	**0.58 (0.14)**	**0.26 (0.21)**	0.42 (0.18)	0.33 (0.25)	**0.01 (0.69)**	**<0.01 (1.00)**	0.13	0.50
	Step Height	0.03 (0.02)	0.04 (0.03)	0.03 (0.02)	0.04 (0.03)	0.36	0.73	1.00	0.36
	Step Length	**0.22 (0.05)**	**0.08 (0.07)**	**0.17 (0.09)**	**0.09 (0.10)**	**0.01 (0.96)**	**0.01 (0.64)**	0.73	**0.05 (0.38)**
B	Lumbar Yaw Range	**3.87 (2.21)**	6.45 (12.76)	**22.95 (9.35)**	**6.65 (9.54)**	0.05	**<0.01 (−1.00)**	0.57	**0.04 (0.78)**
	Trunk Yaw Range	**2.58 (1.18)**	3.68 (3.09)	**22.31 (5.79)**	**6.09 (10.63)**	0.43	**<0.01 (−1.00)**	0.65	**<0.01 (−1.00)**
	Lumbar Yaw Rate	**5.11 (3.41)**	7.63 (11.28)	**21.90 (14.68)**	**7.80 (7.54)**	1.00	**<0.01 (−1.00)**	0.82	**0.04 (0.78)**
	Trunk Yaw Rate	**4.08 (2.26)**	5.92 (4.81)	**20.32 (7.74)**	**7.11 (10.77)**	0.25	**<0.01 (−1.00)**	0.50	**<0.01 (−1.00)**

**Table 5:** Comparisons of gait variability during phases of the walk and turn with obstacle task for controls. *p*-values (*p*) are reported for each comparison with effect sizes (*r*) indicated for significant differences. Comparisons: Pre-turn pre-obstacle to pre-turn post obstacle (pre-turn), pre-turn pre-obstacle to post-turn pre-obstacle (pre-obs), pre-turn post-obstacle to post-turn post-obstacle (post-obs), and post-turn pre-obstacle to post-turn post-obstacle (post-turn).

		Mean (STD) Gait Variability				Pre-Turn	Pre-Obs	Post-Obs	Post-Turn
	Gait Parameter	Pre-Turn Pre-Obstacle	Pre-Turn Post-Obstacle	Post-Turn Pre-Obstacle	Post-Turn Post-Obstacle	*p*(*r*)	*p*(*r*)	*p*(*r*)	*p*(*r*)
T	Cadence	**4.49 (3.89)**	12.90 (13.67)	**9.31 (7.22)**	**5.03 (10.02)**	0.32	**0.01 (−0.73)**	0.12	**0.05 (0.39)**
	Contact Time	**0.06 (0.02)**	**0.09 (0.15)**	0.07 (0.09)	0.07 (0.11)	**0.05 (0.82)**	0.52	0.37	0.17
	Step Time	**0.07 (0.02)**	0.20 (0.23)	**0.12 (0.06)**	**0.06 (0.08)**	0.32	**0.01 (−0.58)**	0.1	**0.04 (0.52)**
S	Foot Attack Angle	24.90 (23.67)	13.29 (14.79)	**37.02 (28.82)**	**11.29 (9.77)**	0.07	1	0.97	**<0.01 (−0.15)**
	Lateral Deviation	**0.02 (0.02)**	0.03 (0.02)	**0.04 (0.02)**	**0.02 (0.02)**	0.83	**0.04 (−0.58)**	0.24	**0.02 (0.39)**
	Max Swing Velocity	0.68 (0.43)	0.44 (0.42)	**0.57 (0.36)**	**0.23 (0.14)**	0.32	0.15	0.76	**0.02 (0.12)**
	Step Height	**0.03 (0.01)**	0.03 (0.02)	**0.04 (0.02)**	**0.03 (0.03)**	0.7	**0.01 (−0.55)**	0.58	**0.01 (0.15)**
	Step Length	0.29 (0.26)	0.16 (0.18)	**0.21 (0.11)**	**0.08 (0.04)**	0.24	0.15	0.9	**0.02 (0.33)**
B	Lumbar Yaw Range	**6.56 (9.11)**	**30.04 (41.23)**	**25.58 (20.99)**	**7.65 (12.57)**	**0.04 (−0.64)**	**0.01 (−0.45)**	**0.04 (0.67)**	**<0.01 (0.67)**
	Trunk Yaw Range	**11.04 (20.78)**	**20.93 (39.19)**	**38.08 (31.41)**	**6.58 (5.92)**	0.64	**<0.01 (−0.70)**	**0.01 (1.00)**	**<0.01 (0.97)**
	Lumbar Yaw Rate	**11.46 (20.36)**	**31.59 (36.54)**	30.28 (21.26)	16.86 (36.60)	**0.01 (−0.55)**	0.17	0.17	0.32
	Trunk Yaw Rate	**17.05 (26.87)**	**19.11 (20.76)**	**44.89 (38.69)**	**9.91 (9.07)**	0.32	**0.01 (−0.79)**	**0.04 (1.00)**	**<0.01 (0.64)**

**Table 6:** Comparisons of gait variability between PwMS (MS) and controls (HC) during each phase of OW task. *p*-values (*p*) are reported for each comparison with effect sizes (*r*) indicated for significant differences.

		Pre-Turn Pre-Obstacle			Pre-Turn Post-Obstacle			Post-Turn Pre-Obstacle			Post-Turn Post-Obstacle		
	Gait Parameter	MS Mean (STD)	HC Mean (STD)	*p*(*r*)	MS Mean (STD)	HC Mean (STD)	*p*(*r*)	MS Mean (STD)	HC Mean (STD)	*p*(*r*)	MS Mean (STD)	HC Mean (STD)	*p*(*r*)
T	Cadence	5.27 (1.44)	4.66 (4.40)	0.25	3.66 (5.57)	12.00 (14.00)	0.38	5.97 (3.99)	7.30 (5.32)	0.38	2.48 (1.95)	2.28 (2.52)	0.74
	Contact Time	0.11 (0.03)	0.06 (0.02)	0.01 (1.00)	0.03 (0.02)	0.11 (17)	0.74	0.12 (0.10)	0.08 (0.10)	0.25	0.08 (0.15)	0.09 (0.13)	0.84
	Step Time	0.11 (0.04)	0.07 (0.02)	0.05	0.045 (0.03)	0.21 (0.26)	0.25	0.12 (0.07)	0.12 (0.06)	0.84	0.05 (0.04)	0.04 (0.06)	0.74
S	Foot Attack Ang.	52.37 (35.87)	20.32 (17.64)	0.25	20.40 (21.86)	10.21 (9.13)	0.31	39.34 (37.57)	35.04 (21.96)	0.94	30.85 (43.19)	9.90 (9.66)	0.25
	Lateral Deviation	0.02 (0.01)	0.02 (0.02)	0.64	0.03 (0.01)	0.03 (0.02)	0.46	0.03 (0.02)	0.04 (0.03)	0.25	0.02 (0.01)	0.02 (0.02)	0.64
	Max Swing Vel.	0.57 (0.14)	0.69 (0.48)	0.95	0.26 (0.23)	0.44 (0.38)	0.55	0.41 (0.17)	0.50 (39)	0.64	0.34 (0.25)	0.26 (0.14)	0.84
	Step Height	0.03 (0.02)	0.03 (0.02)	0.84	0.04 (0.03)	0.03 (0.02)	0.74	0.03 (0.02)	0.04 (0.02)	0.05 (−0.89)	0.04 (0.03)	0.03 (0.03)	0.64
	Step Length	0.22 (0.06)	0.30 (0.29)	0.46	0.08 (0.08)	0.16 (0.17)	0.2	0.15 (0.08)	0.20 (0.11)	0.38	0.09 (0.10)	0.09 (0.04)	0.55
B	Lum. Yaw Range	4.13 (2.20)	8.32 (10.19)	0.74	7.08 (13.44)	35.00 (45.56)	0.08	21.00 (7.23)	26.96 (24.16)	1	7.23 (9.99)	6.88 (11.50)	0.84
	Trunk Yaw Range	2.66 (1.22)	14.5 (23.60)	0.05	3.89 (3.22)	23.23 (44.79)	0.08	22.27 (5.73)	43.42 (35.07)	0.38	6.62 (11.19)	5.76 (5.20)	0.95
	Lum. Yaw Rate	5.02 (3.44)	14.22 (23.41)	0.84	**8.29 (11.82)**	**37.09 (39.86)**	**0.04 (−0.33)**	18.32 (7.51)	29.50 (21.95)	0.74	8.35 (7.82)	19.14 (42.76)	0.74
	Trunk Yaw Rate	3.85 (2.13)	22.06 (30.19)	0.05	**6.36 (4.92)**	**20.84 (23.33)**	**0.04 (−0.67)**	18.73 (6.10)	51.13 (41.89)	0.08	7.78 (11.27)	7.78 (5.65)	0.84

## Data Availability

The data presented in this study are available on request from the corresponding author. The data are not publicly available due to Institutional Review Board limitations.
